# Supplementary figures and images for: Prediction of future aging-related slow gait and its determinants with deep learning and logistic regression
Source: PLoS One. 2025 Jun 17;20(6):e0325172. doi: 10.1371/journal.pone.0325172 (PMC12173421; doi:10.1371/journal.pone.0325172)

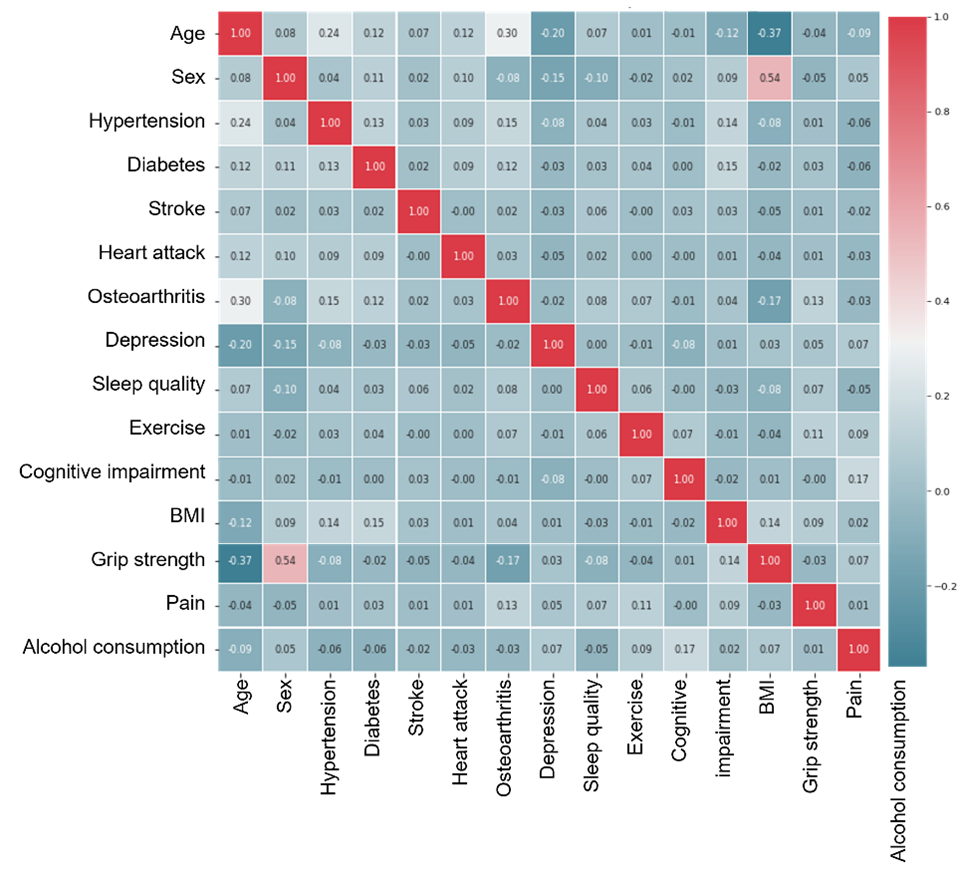

Supplement: S1 Fig — The scale bar on the right shows the color coding from red (strongly positively correlated) to blue (strongly negatively correlated). (TIF) [file pone.0325172.s001.tif]

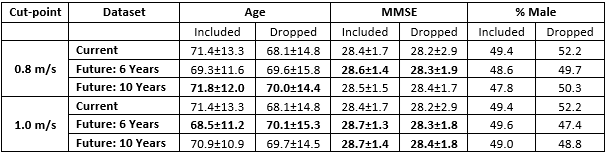

Supplement: S1 Table — Values are median + /- standard deviation. Bold text indicates values that were found to be significantly different. (TIF) [file pone.0325172.s002.tif]

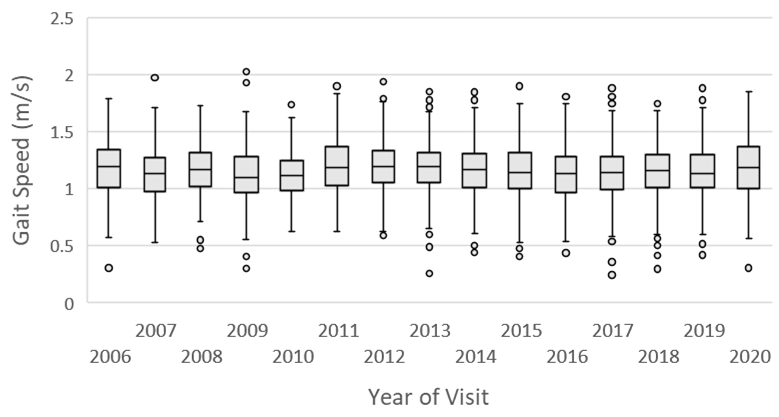

Supplement: S2 Fig — The lack of a clear trend across the means suggests minimal dataset drift over the year of visit. (TIF) [file pone.0325172.s003.tif]

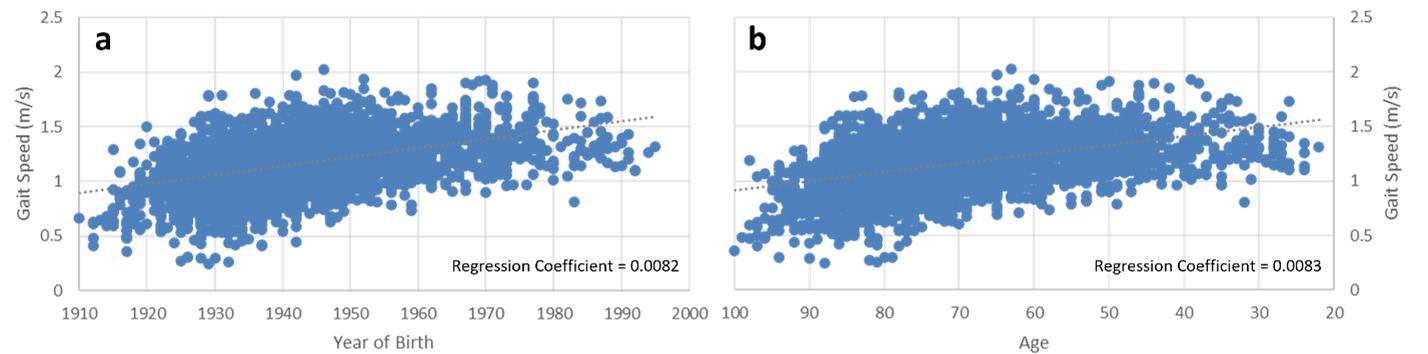

Supplement: S3 Fig — a) Analysis of potential dataset drift in the year of birth for all BLSA subjects used in this study. The dotted line indicates a linear regression fit with regression coefficient of 0.0082. b) Comparison to a regression fit of age versus gait speed (decreasing left to right) with coefficient of 0.0083. The similar coefficients and shape of the data indicate minimal dataset drift due to year of birth. (TIF) [file pone.0325172.s004.tif]

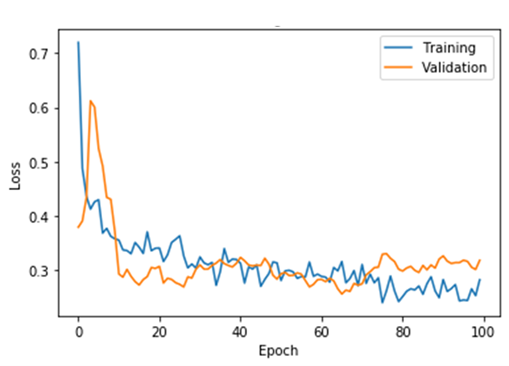

Supplement: S4 Fig — The blue line indicates the loss of the training set, and the orange line represents the loss of the validation set. These loss curves show minimal overfitting after hyperparameter tuning. (TIF) [file pone.0325172.s005.tif]

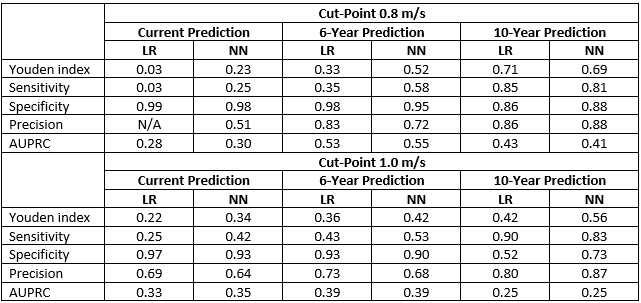

Supplement: S2 Table — The AUPRC value is the difference between the AUC and the no-skill AUC. (TIF) [file pone.0325172.s006.tif]

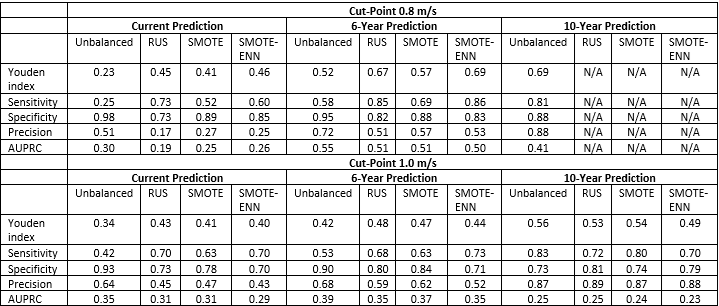

Supplement: S3 Table — The AUPRC value is the difference between the AUC and the no-skill AUC. RUS = Random Undersampling, SMOTE = Synthetic Minority Oversampling Technique, ENN = Edited Nearest Neighbors. The asterisk indicates the classifier that did not need class balancing techniques because the data was already balanced. (TIF) [file pone.0325172.s007.tif]

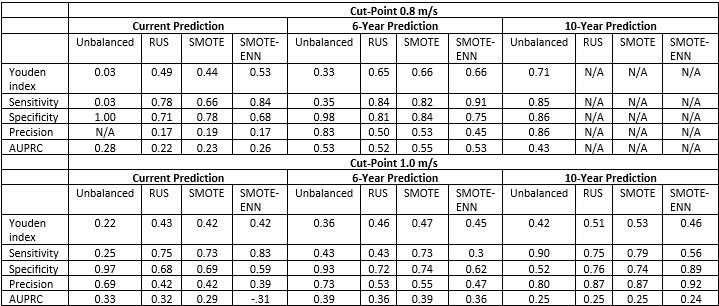

Supplement: S4 Table — The AUPRC value is the difference between the AUC and the no-skill AUC. RUS = Random Undersampling, SMOTE = Synthetic Minority Oversampling Technique, ENN = Edited Nearest Neighbors. The asterisk indicates the classifier that did not need class balancing techniques because the data was already balanced. (TIF) [file pone.0325172.s008.tif]

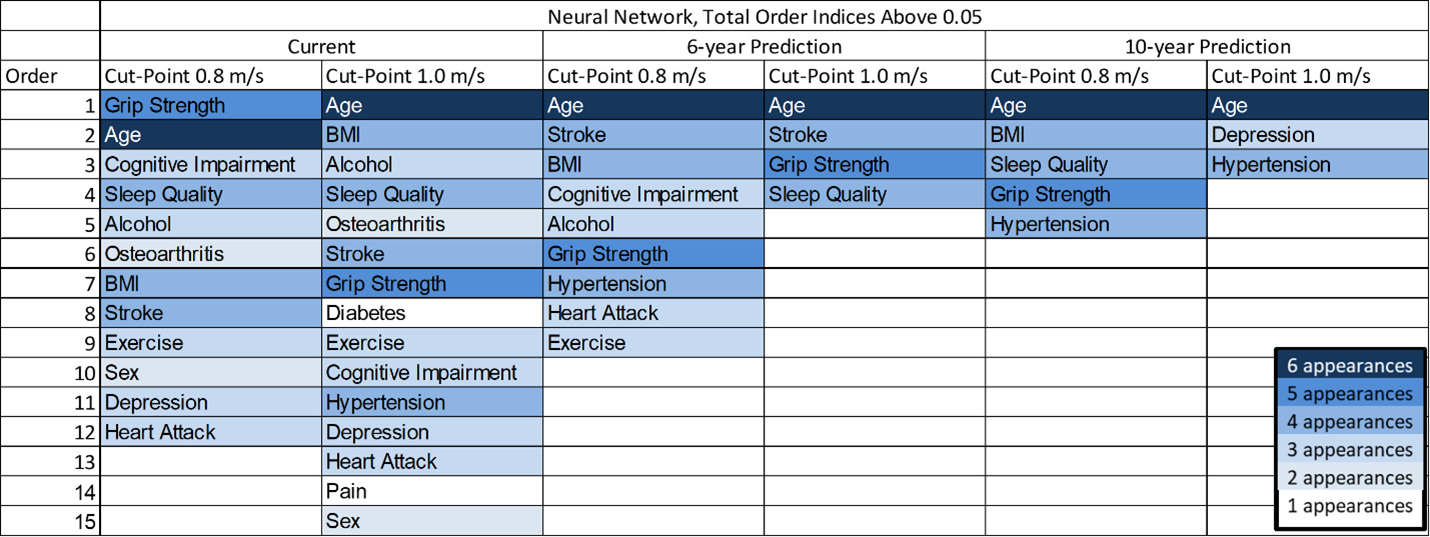

Supplement: S5 Fig — The color scale is shown in the inset, where darker colored cells indicate variables that are found to be significant more frequently across classifiers. (TIF) [file pone.0325172.s009.tif]

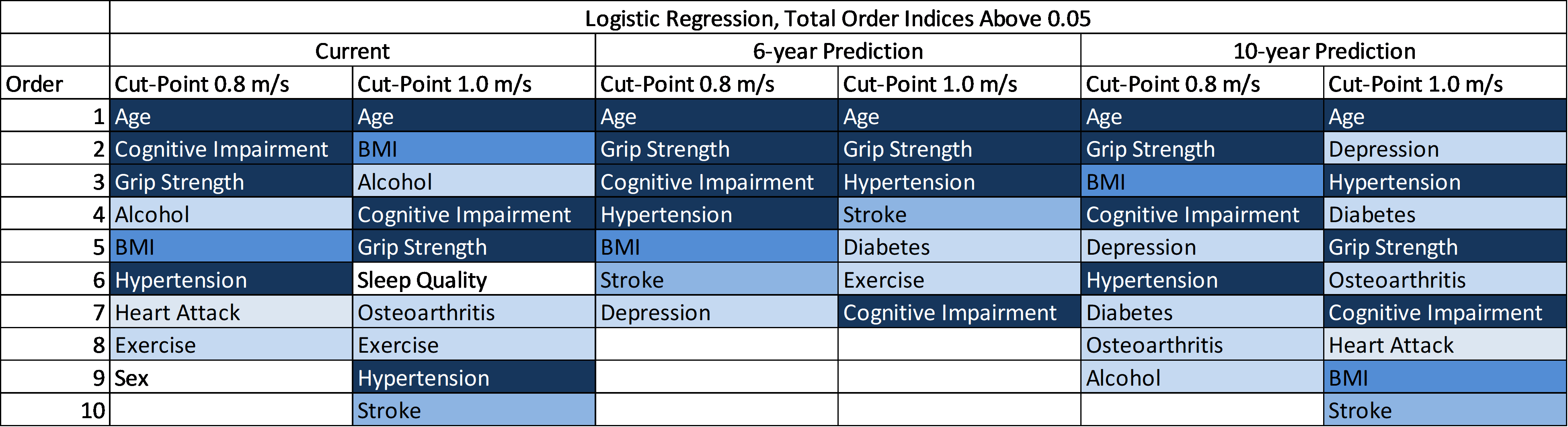

Supplement: S6 Fig — The color scale is shown in the inset in S5 Fig, where darker colored cells indicate variables that are found to be significant more frequently across classifiers. (TIF) [file pone.0325172.s010.tif]

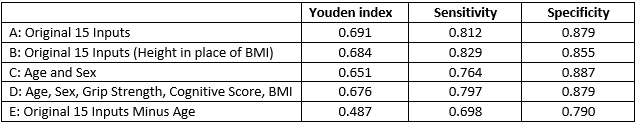

Supplement: S5 Table — A: 15 Original inputs, B: Original inputs with height in place of BMI, C: Age and sex alone, D: Age, sex, BMI, grip strength, cognitive score (all the quantitative original inputs) E: Original inputs except age. (TIF) [file pone.0325172.s011.tif]
