# Supplementary material for: Prediction of future aging-related slow gait and its determinants with deep learning and logistic regression
Source: PLoS One. 2025 Jun 17;20(6):e0325172. doi: 10.1371/journal.pone.0325172 (PMC12173421; doi:10.1371/journal.pone.0325172)
Supplement: Attachment — Submitted filename: Review Form.docx [file pone.0325172.s012.docx]

Prediction of future aging-related slow gait and its determinants with deep learning and

logistic regression

**Intro**

1. The authors highlight the need for larger datasets in deep learning analyses and provide sample sizes from previous studies (108, 239, 746, 1901) as examples. However, no references are provided to support these figures. Including citations for these studies would enhance transparency and allow readers to verify the source of this information. (Line 81-86)
2. The introduction would benefit from a clearer explanation of how the present study differs from previous research and which specific gap in the literature it aims to address. For instance, the statement 'Several attempts have been made using statistical models to predict gait speed changes from a narrow set of potential predictors' suggests prior work in this area. However, it would be helpful if the authors explicitly outlined how their study expands upon or improves these past efforts. Additionally, specifying the professional groups (health workers, physiotherapist, physician etc.) that could benefit from these findings would provide readers with a clearer understanding of the study’s relevance and impact.

**Methods**

3. The primary aim of this study appears to focus on the relationship between gait speed, aging, and mortality. However, gait speed can be influenced by a wide range of factors, including orthopedic, neurological, and other medical conditions. Wouldn't it be more informative to stratify the analysis by different subgroups to account for these variations? Discussing the potential impact of such factors and whether a subgroup analysis could enhance the findings would strengthen the study's interpretation and applicability.
4. I acknowledge that biostatistics and methodological details can sometimes be complex to fully follow, so I apologize if I have missed anything. That being said, I would like to raise a few points regarding the chosen cut-points and the study population. The authors have established clinically relevant cut-points for gait speed (0.8 m/s and 1.0 m/s) based on prior literature. However, gait speed thresholds may vary across different populations, particularly in individuals with neurological conditions such as Parkinson’s disease or stroke. Were disease-specific variations in cut-points considered when applying these thresholds to the study population? If not, this could be a limitation, as the same cut-points may not be appropriate for all individuals.
5. Additionally, the study utilized gait speed data from the BLSA cohort, where measurement intervals varied by age. Since older individuals had more frequent assessments, could this have biased the long-term predictive model? Were adjustments made to account for the potential overrepresentation of gait speed decline in older participants? A discussion on how these factors may have influenced the results would strengthen the study’s findings.
6. It would be better for the readers if the authors explained a point about the measurement and use of grip strength in their analysis. In the table describing input variables, grip strength is reported in kilograms (Table 2). However, it is specifically labeled as "hand grip muscles right (kg)." Could you clarify whether only the right hand was measured and analyzed? If so, what was the rationale for not including the left hand or using an alternative approach such as the mean of both hands or the dominant hand? Additionally, considering that grip strength was identified as a significant factor in the Sobol index analysis and highlighted as a basis for therapeutic intervention, do you believe that using only the right hand impacts the generalizability of your findings? If left-hand data were available, would incorporating it alter your results? Clarifying these points would strengthen the methodological transparency and the applicability of your findings.
7. Previous studies (e.g., Sadeghi et al., 2000) suggest that lower limb dominance plays a role in gait biomechanics, with the dominant limb often contributing more to propulsion while the non-dominant limb provides stability. Considering this, do you think assessing lower limb dominance could enhance the interpretation of gait speed determinants in your study?

**Discussion**

8. The discussion would benefit from a more detailed justification of why grip strength was emphasized as a key predictor of gait speed rather than more directly related lower-limb strength measures. While grip strength has been associated with overall strength and function, gait speed is likely more directly influenced by lower-limb muscle strength. Addressing this distinction and discussing potential reasons for the focus on grip strength over leg strength measures would provide greater clarity for readers.
9. The authors have considered multiple gait-related parameters in their predictive model. However, in clinical and health-related research, patient-reported outcome measures (PROMs) are frequently used to capture the patient's perspective. PROMs can provide valuable insight into how individuals perceive their mobility, fatigue, pain, or fear of falling, which are factors that could influence gait patterns over time. Did the authors consider incorporating PROMs while examining the determinants of slow gait? If not, this could be a potential limitation of the study, as relying solely on objective gait parameters may overlook important subjective experiences that contribute to mobility decline. Including PROMs in future research could enhance the model's generalizability and provide a more comprehensive understanding of aging-related gait changes.
